# Supplementary material for: Varying demands for cognitive control reveals shared neural processes supporting semantic and episodic memory retrieval
Source: Nat Commun. 2021 Apr 9;12:2134. doi: 10.1038/s41467-021-22443-2 (PMC8035200; doi:10.1038/s41467-021-22443-2)
Supplement: Supplementary file 1 — Supplementary Information [file 41467_2021_22443_MOESM1_ESM.pdf]

Supplementary Information: Varying demands for cognitive control reveals shared neural processes supporting semantic and episodic memory retrieval

Deniz Vatansever[1,2*], Jonathan Smallwood[2], Elizabeth Jefferies[2]

[1] Institute of Science and Technology for Brain-inspired Intelligence, Fudan University, Shanghai, PR China, 200433
[2] Department of Psychology, University of York, York, United Kingdom, YO10 5DD

*Address for correspondence:
Deniz Vatansever, PhD
Institute of Science and Technology for Brain-inspired Intelligence
Fudan University
220 Handan Road, East Guanghua Building
Shanghai, PR China, 200433
Tel: +86 021 6564 7645
E-mail: deniz@fudan.edu.cn

Table of Contents

SUPPLEMENTARY NOTES **2**

SECTION S1. BEHAVIOURAL PERFORMANCE ACROSS 3-AFC FMRI TASKS 2
SECTION S2. MEASUREMENTS OF FLUID INTELLIGENCE AND SUSTAINED ATTENTION 2

SUPPLEMENTARY FIGURES **4**

FIGURE S1. MANIPULATION OF SEMANTIC ASSOCIATIVE STRENGTH 4
FIGURE S2. PSYCHOLINGUISTIC PROPERTIES OF THE FMRI TASK STIMULI 5
FIGURE S3. ADDITIONAL EFFECTS OF TASK DIFFICULTY ON NEURAL RESPONSES 6
FIGURE S4. SHARED PROCESSES IN THE AUTOMATIC RETRIEVAL OF LONG-TERM MEMORY 7
FIGURE S5. GROUP-LEVEL INTRINSIC CONNECTIVITY OF THE LIFG/AINS CLUSTER 8
FIGURE S6. DIFFERENTIAL NEURAL CIRCUITS RELATED TO LONG-TERM MEMORY RETRIEVAL 9
FIGURE S7. FMRI DATA QUALITY ASSESSMENT ACROSS EXPERIMENTS 10

Supplementary Notes

Section S1. Behavioural performance across 3-AFC fMRI tasks

Our initial objective was to assess the behavioural output of participants during the performance of the two 3-AFC fMRI tasks that were designed to probe two different memory types under two levels of memory strength manipulation. As expected, paired sample t-tests (corrected for multiple comparisons) revealed significantly better performance for strong versus weak associations, on both the semantic and episodic 3-AFC fMRI tasks. Within semantic memory retrieval, the average inverse efficiency score (i.e. response latency weighted by the number of incorrect responses) for the strong association trials was significantly lower than that of weak trials (1753.27 ± 55.39 ms vs. 2223.40 ± 56.95 ms, $t_{(45)} = -14.09$, $p < .001$), thus indicating better performance. Further analysis revealed significantly lower latency to correct responses (1674.67 ± 49.49 ms vs. 2026.51 ± 43.64 ms, $t_{(45)} = -14.31$, $p < .001$) as well as higher percentage of correct responses (95.87 ± .58 % vs. 91.69 ± .76 %, $t_{(45)} = 5.44$, $p < .001$) in the strong versus weak trials. A similar pattern followed for episodic memory retrieval. The mean inverse efficiency score for the strong association trials was significantly lower than that of weak trials (1483.07 ± 42.06 ms vs. 1650.21 ± 55.74 ms, $t_{(45)} = -4.94$, $p < .001$). Further analysis revealed significantly smaller latency for correct responses on strong compared with weak episodic trials (1466.67 ± 38.33 ms vs. 1603.13 ± 41.44 ms, $t_{(45)} = -6.02$, $p < .001$), yet no significant difference was observed in the percentage of correct responses for this strength manipulation (99.08 ± .33 vs. 98.04 ± .68, $t_{(45)} = 1.64$, $p = .11$) (Fig. 2a-c).

When directly comparing semantic and episodic memory retrieval, participants performed significantly better in the episodic than in the semantic 3-AFC task, with lower mean inverse efficiency score (1563.06 ms ± 45.39 vs. 1976.60 ± 53.25 ms, $t_{(45)} = 7.64$, $p < .001$), smaller latency for correct responses (1533.57 ± 38.01 ms vs. 1846.44 ± 44.91 ms, $t_{(45)} = 7.06$, $p < .001$) and higher percentage of correct responses (98.56 ± .43 % vs. 93.77 ± .56 %, $t_{(45)} = -7.77$, $p < .001$).

Section S2. Measurements of fluid intelligence and sustained attention

With the aim of assessing fluid intelligence and educative ability, all participants in Experiment 2 completed the Raven's Advanced Progressive Matrices (RAPM). This task required participants to identify patterns amongst 8 boxes of stimuli presented in a 3 by 3 matrix layout (the 9th box did not contain a pattern) presented on top of the screen. An additional 4 boxes with unique patterns were presented at the bottom of the screen. The participants were required to select one of 4 boxes that could replace the empty box based on the relationship amongst the presented stimuli. The test was presented in two phases: (i) a practice phase with 2 problem sets and (ii) a test phase with 36 problem sets. Corrective feedback was provided to the participants during the practice phase. However, no feedback was given during the test phase in which the participants were required to complete as many problems as possible within 20 minutes. Difficulty of the problems increased with each trial. The proportion of correct trials out of the number of attempted trials was employed as an index of performance in this task (mean = 67 ± 17 %). There was no significant correlation between

LIFG/aINS to vmPFC connectivity and the proportion of correct trials out of the number of trials attempted in the RAPM task (partial r_p = -.010, p = .45, two-tailed, corrected for age, gender and in-scanner head motion).

In order to assess selective attention and inhibitory control, all participants in Experiment 2 completed the Flanker task. In this task, participants were presented with a target arrowhead at the centre of the screen in white font on a black background, pointing either to the left or the right, which was flanked by 2 to 4 items on either side. The flankers were arrowheads either in the same direction as the target (congruent condition), opposite direction to the target (incongruent condition), or were replaced with lines instead of arrowheads (neutral condition). In each trial, participants were asked to press either left or right buttons based on the direction of the target, while ignoring the flanker items. Following a fixation period that lasted between 900-2100 ms, the targets and flankers appeared, and the participants were allowed 1700 ms to respond. Subsequently, a post-target fixation was presented lasting until the end of the 4000 ms total trial period. A total of 72 trials were presented, which were divided into three sets of 24 incongruent, congruent and neutral trials (further divided into two sets of 12 left and right directions and three sets of two, three, and four Flankers). Since all participants showed high accuracy (above 80%) in the incongruent condition (mean = 94.94 ± 9.19 %), the reaction time to incongruent condition was used as an index of performance in this task (mean = 537.28 ± 90.66 ms). There was no significant correlation between LIFG/aINS to vmPFC connectivity and the average reaction time to incongruent trials in the Flanker task (partial r_p = -.052, p = .27, two-tailed, corrected for age, gender and in-scanner head motion).

Supplementary Figures

Figure S1. Manipulation of semantic associative strength

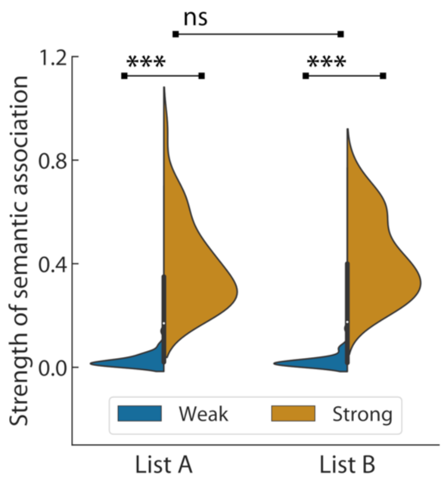

Figure S1. Manipulation of semantic associative strength. The semantic associative strength between the probe and target word-pairs was manipulated based on the Edinburgh Associative Thesaurus (http://rali.iro.umontreal.ca/word-associations/query/). Two lists of word-pairs were used that were randomized across participants. In list A, the strength of association between the probe and target word-pairs was significantly lower in the weak (.028 ± .028) as compared to the strong association trials (.39 ± .17) ($t_{(2,78)}$ = -13.41, p < .001). Similarly in list B, the strength of association between the probe and target word-pairs was significantly lower in the weak (.025 ± .028) as compared to the strong association trials (.42 ± .16) ($t_{(2,78)}$ = -15.64, p < .001). However, there was no statistically significant difference in the mean strength of all trials between list A (.21 ± .22) and list B (.22 ± .23) ($t_{(2,158)}$ = -.38, p = .71). The violin plots illustrate a boxplot with the median (centre white dot), the interquartile range (black bar), the minima/maxima values (thin black line) as well as the kernel density estimation of the underlying distribution. *** denotes p < 0.001. Paired t-tests were employed, corrected for multiple comparisons. Data include n = 40 independent word-pairs for each stimulus category (strong and weak) in each word list (List A and List B). Source data are provided as a Source Data file.

Figure S2. Psycholinguistic properties of the fMRI task stimuli

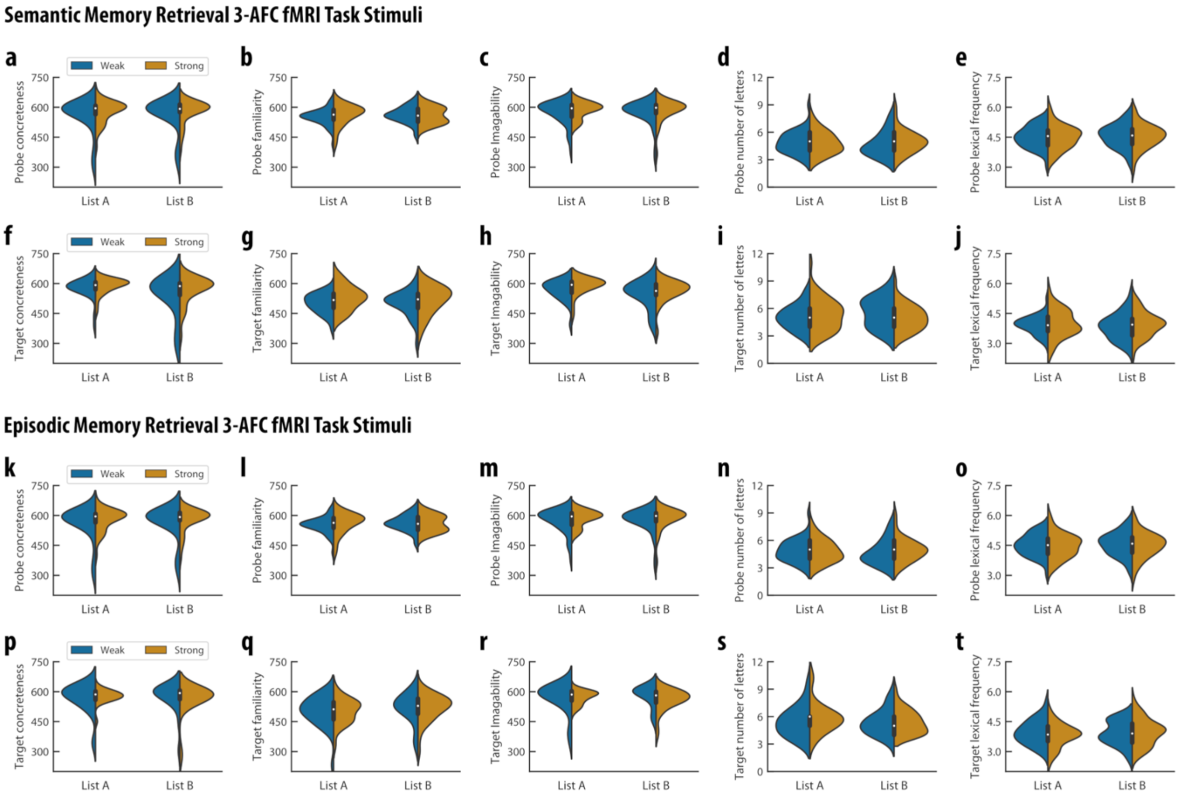

Figure S2. Psycholinguistic properties of the fMRI task stimuli. All words employed in the 3-AFC memory tasks were chosen from a database based on our prior investigations, matched for common psycholinguistic properties. While concreteness, familiarity, imageability and number of letters were obtained from the MRC psycholinguistic database (https://websites.psychology.uwa.edu.au/school/MRCDatabase/uwa_mrc.htm), the lexical frequency was extracted from the SUBLEX-UK database (http://crr.ugent.be/archives/1423). The violin plots illustrate the kernel density estimation of the underlying distribution across five psycholinguistic properties of the probe and target words across trial types, word lists and task types, whereas the boxplots depict the median (centre white dot), the interquartile range (black bar) and the minima/ maxima values (thin black line). Multivariate general linear models revealed no significant difference in the psycholinguistic properties (i.e. concreteness, familiarity, imageability, word length, lexical frequency) of the probe and target words within the 3-AFC fMRI tasks, based either on memory type (semantic versus episodic memory, $F_{(5, 484)} = .38$, $p = .87$; Wilk's $\Lambda = .99$, partial $\eta^2 = .004$), memory strength (strong versus weak associations, $F_{(5, 484)} = 1.33$, $p = .25$; Wilk's $\Lambda = .99$, partial $\eta^2 = .014$), or list (list A versus list B, $F_{(5, 484)} = .57$, $p = .77$; Wilk's $\Lambda = .99$, partial $\eta^2 = .006$). Data include n = 40 independent word-pairs for each stimulus category (strong and weak) and each word list (List A and List B) across the two fMRI tasks (semantic and episodic). Source data are provided as a Source Data file.

Figure S3. Additional effects of task difficulty on neural responses

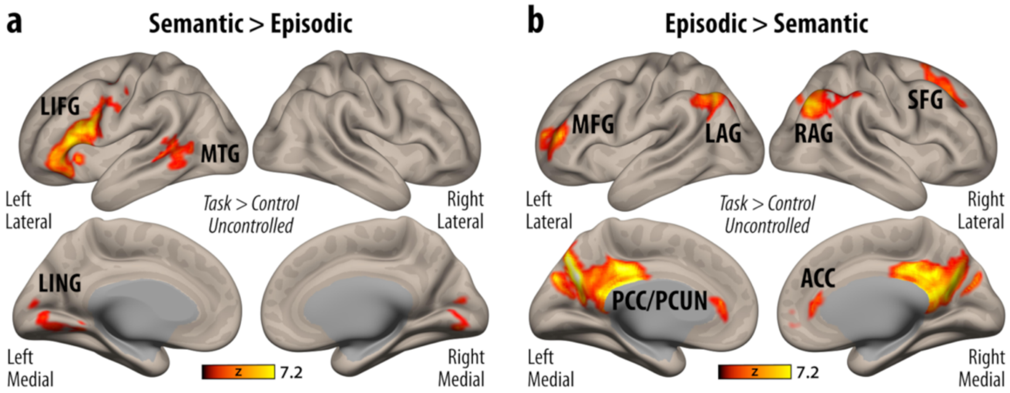

Figure S3. Additional effects of task difficulty on neural responses. Given performance differences observed across memory types, the main fMRI analysis reported in the manuscript was controlled for inverse efficiency score in order to account for potential task difficulty effects. Above we report the uncontrolled version of this analysis. **(a)** In comparison to the controlled version, the semantic > episodic contrast displayed additional effects centred on the middle temporal (MTG) and lingual gyri. **(b)** The reverse contrast on the other hand, illustrated additional effects across the superior (SFG) and middle frontal gyri (MFG) as well as the anterior cingulate cortex (ACC). All results were multiple comparison corrected using the family-wise error (FWE) detection technique at the 0.05 level of significance (z = 2.6).

Figure S4. Shared processes in the automatic retrieval of long-term memory

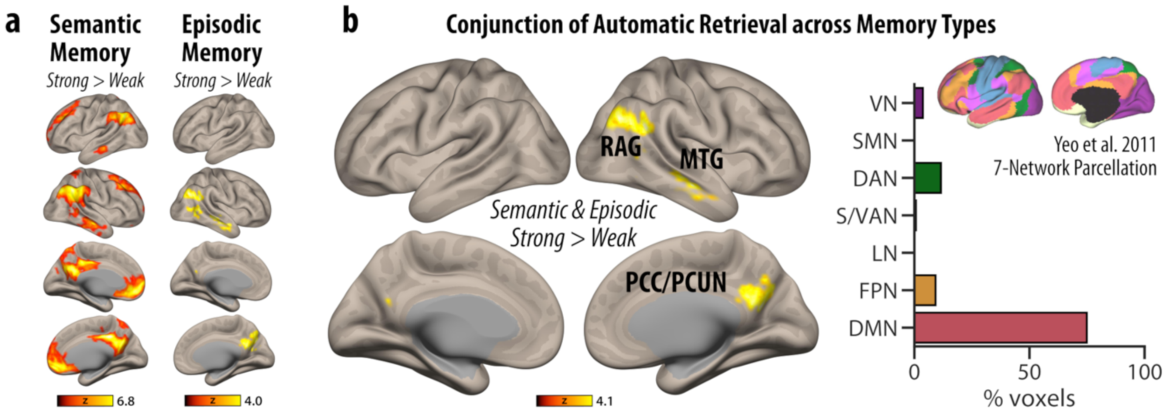

Figure S4. Shared processes in the automatic retrieval of long-term memory. In addition to controlled retrieval investigated in the main study, we further interrogated any commonalities in the neural responses for the retrieval of strong versus weak associations across memory types. **(a)** For both semantic and episodic 3-AFC fMRI tasks, shared neural responses were observed for the retrieval of strongly versus weakly associated word-pairs. **(b)** A formal conjunction analysis illustrated that for the strong > weak contrast, the posterior cingulate/precuneal cortices (PCC/PCUN), right angular (RAG) and middle temporal gyri (MTG) showed greater activity for both memory types, which almost exclusively overlapped with the default mode network (based on the Yeo 7-Network parcellation) (VN = visual network, SMN = somatomotor network, DAN = dorsal attention network, S/VAN = salience/ventral attention network, LN = limbic network, FPN = frontoparietal network, DMN = default mode network). All results were multiple comparison corrected using the family-wise error (FWE) detection technique at the 0.05 level of significance (z = 2.6).

Figure S5. Group-level intrinsic connectivity of the LIFG/aINS cluster

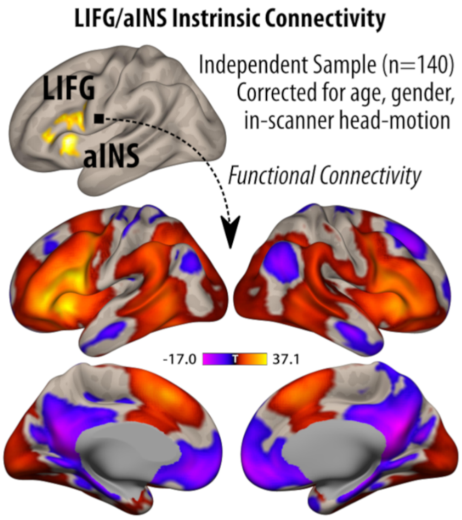

Figure S5. Group-level intrinsic connectivity of the LIFG/aINS cluster. The cluster which showed shared activity in weak versus strong trials across semantic and episodic 3-AFC tasks in Experiment 1 was used as a region-of-interest in a seed-based functional connectivity analysis (LIFG = left inferior frontal gyrus, aINS = anterior insular cortex). Across the whole group of 140 participants, a one-sample t-test (correcting for age, gender and in-scanner head motion) revealed widespread connectivity to both transmodal and unimodal brain regions at rest. While positive connectivity was observed to regions implicated in the frontoparietal, salience/ventral/dorsal attention networks as well as the visual network, negative connectivity (anti-correlations) spanned regions of the default mode and somatomotor networks (based on the Yeo-7 Network parcellation scheme). Reported clusters were corrected for multiple comparisons using the family-wise error (FWE) detection technique at the 0.05 level of significance (uncorrected at the voxel-level, 0.005 level of significance).

Figure S6. Differential neural circuits related to long-term memory retrieval

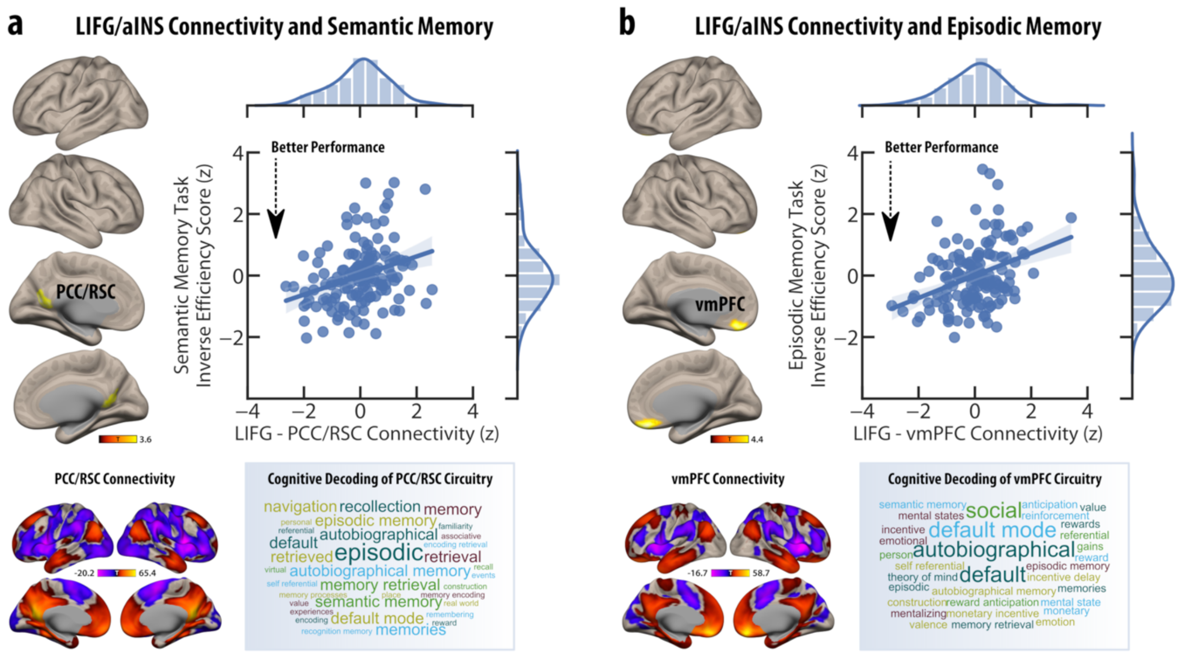

Figure S6. Differential neural circuits related to long-term memory retrieval. Two separate linear regressions were carried out in order to investigate neural circuits that were differentially associated with performance differences across the retrieval of weakly associated semantic and episodic memory. Across participants, **(a)** while reduced positive connectivity (or greater anti-correlation) of the left inferior frontal gyrus and anterior insular cortex (LIFG/aINS) cluster to the bilateral posterior cingulate/retrosplenial cortices (PCC/RSC) was associated with better performance (lower inverse efficiency score) in the retrieval of weak semantic memories, **(b)** reduced connectivity of the same seed region to the ventromedial prefrontal cortex (vmPFC) was linked to selective advantage in the retrieval of weak episodic memories. Additional seed-based connectivity of the PCC/RSC and vmPFC clusters identified largely overlapping connectivity profiles that were centred on the default mode network. Reported clusters were corrected for multiple comparisons using the family-wise error (FWE) detection technique at the 0.05 level of significance (uncorrected at the voxel-level, 0.005 level of significance). In addition, the meta-analytic decoding of the LIFG/aINS – PCC/RSC and LIFG/aINS – vmPFC revealed comparable terms that spanned "recollection, semantic, episodic and autobiographical memory" as well as distinct terms such as "navigation and incentive delay". While straight lines represent the best linear fit over individual values, shaded areas illustrate 95% confidence intervals (shown for illustrative purposes only). Source data are provided as a Source Data file.

Figure S7. fMRI data quality assessment across experiments

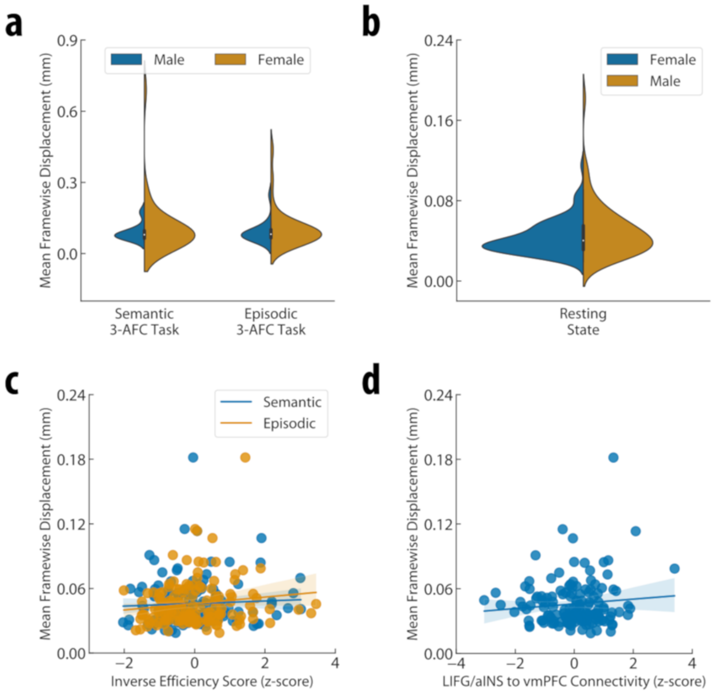

Figure S7. fMRI data quality assessment across experiments. Strict motion-correction procedures were followed across both fMRI tasks and resting state fMRI. In order to further test the potential influence of in-scanner head motion on subsequent analyses, mean framewise displacement score was calculated using the Jenkinson formulation for all participants and MRI scanning sessions. **(a)** The violin plots depict the kernel density estimation of the underlying distribution of framewise displacement across genders and 3-AFC memory retrieval fMRI tasks, whereas the boxplots depict the median (centre white dot), the interquartile range (black bar) and the minima/ maxima values (thin black line). No significant difference was observed in the mean framewise displacement across semantic (.10 ± .098) and episodic memory tasks (.10 ± .071) ($t_{(2,78)}$ = .13, p = .68). n = 46 independent participants examined over two paired fMRI tasks. **(b)** The mean framewise displacement for the resting state fMRI scanning across participants was 0.046 ± .022 mm. There were no significant correlations observed with **(c)** either the inverse efficiency scores across semantic (r = .054, p =.53, two-tailed) and episodic (r = .14, p = .11, two-tailed) memory tasks, or **(d)** the functional connectivity of the identified left inferior frontal gyrus and anterior insular cortex (LIFG/aINS) cluster (r = .10, p = .24, two-tailed). Straight lines represent the best linear fit over individual values, while shared areas illustrate 95% confidence intervals. n = 140 independent participants examined over one experiment. Source data are provided as a Source Data file.
