# Supplementary material for: Varying demands for cognitive control reveals shared neural processes supporting semantic and episodic memory retrieval
Source: Nat Commun. 2021 Apr 9;12:2134. doi: 10.1038/s41467-021-22443-2 (PMC8035200; doi:10.1038/s41467-021-22443-2)
Supplement: Supplementary file 3 — Reporting Summary [file 41467_2021_22443_MOESM3_ESM.pdf]

nature research

Corresponding author(s): Deniz Vatansever

Last updated by author(s): Jan 19, 2021

Reporting Summary

Nature Research wishes to improve the reproducibility of the work that we publish. This form provides structure for consistency and transparency in reporting. For further information on Nature Research policies, see our Editorial Policies and the Editorial Policy Checklist.

Statistics

For all statistical analyses, confirm that the following items are present in the figure legend, table legend, main text, or Methods section.

n/a	Confirmed	
☐	☒	The exact sample size (n) for each experimental group/condition, given as a discrete number and unit of measurement
☐	☒	A statement on whether measurements were taken from distinct samples or whether the same sample was measured repeatedly
☐	☒	The statistical test(s) used AND whether they are one- or two-sided<br>*Only common tests should be described solely by name; describe more complex techniques in the Methods section.*
☐	☒	A description of all covariates tested
☐	☒	A description of any assumptions or corrections, such as tests of normality and adjustment for multiple comparisons
☐	☒	A full description of the statistical parameters including central tendency (e.g. means) or other basic estimates (e.g. regression coefficient) AND variation (e.g. standard deviation) or associated estimates of uncertainty (e.g. confidence intervals)
☐	☒	For null hypothesis testing, the test statistic (e.g. F, t, r) with confidence intervals, effect sizes, degrees of freedom and P value noted<br>*Give P values as exact values whenever suitable.*
☒	☐	For Bayesian analysis, information on the choice of priors and Markov chain Monte Carlo settings
☒	☐	For hierarchical and complex designs, identification of the appropriate level for tests and full reporting of outcomes
☒	☐	Estimates of effect sizes (e.g. Cohen's d, Pearson's r), indicating how they were calculated

Our web collection on statistics for biologists contains articles on many of the points above.

Software and code

Policy information about availability of computer code

Data collection	PsychoPy2 (Version 1.82) was employed for behavioural data collection at the laboratory and for stimulus delivery inside the MRI scanner. A 3T GE HDx Excite scanner was used for the collection of MRI data.
Data analysis	FSL (Version 5.0.11), FSL FEAT (Version 6.0), FSL FLIRT (Version 6.0), SPM (Version 12.0), MATLAB (Version 16.a) and CONN Toolbox (Version 17.f) were employed for data analysis.

For manuscripts utilizing custom algorithms or software that are central to the research but not yet described in published literature, software must be made available to editors and reviewers. We strongly encourage code deposition in a community repository (e.g. GitHub). See the Nature Research guidelines for submitting code & software for further information.

Data

Policy information about availability of data

All manuscripts must include a data availability statement. This statement should provide the following information, where applicable:

- Accession codes, unique identifiers, or web links for publicly available datasets
- A list of figures that have associated raw data
- A description of any restrictions on data availability

The datasets generated and/or analysed during the current study are not publicly available due to institutional regulations, ethics and confidentiality agreements, but are available from the corresponding author on reasonable request. Unthresholded statistical maps (z-maps) from the task-based and resting-state fMRI portions of this study are publicly available at https://identifiers.org/neurovault.collection:8431. Additionally, source data are provided with this paper.

Field-specific reporting

Please select the one below that is the best fit for your research. If you are not sure, read the appropriate sections before making your selection.

☒ Life sciences ☐ Behavioural & social sciences ☐ Ecological, evolutionary & environmental sciences

For a reference copy of the document with all sections, see nature.com/documents/nr-reporting-summary-flat.pdf

Life sciences study design

All studies must disclose on these points even when the disclosure is negative.

Sample size	In total, 47 undergraduate or postgraduate students were recruited for Experiment 1 and 169 students were recruited for Experiment 2. These sample sizes were chosen as relatively large samples for fMRI research based on prior literature on related topics (Lanzoni et al. 2019; Turnbull et al. 2019).
Data exclusions	In Experiment 1, one participant had incomplete data which was excluded from further analysis. In Experiment 2, a total of 29 participants were excluded due to missing data and excessive motion inside the scanner based on the extensive head-motion correction procedures employed in this study that were pre-defined.
Replication	There were no direct replication attempts performed in this study, as the design and sample size are unique and relatively large.
Randomization	There were no between group analyses conduced in this study.
Blinding	There were no between group analyses conducted in this study.

Reporting for specific materials, systems and methods

We require information from authors about some types of materials, experimental systems and methods used in many studies. Here, indicate whether each material, system or method listed is relevant to your study. If you are not sure if a list item applies to your research, read the appropriate section before selecting a response.

Materials & experimental systems

n/a	Involved in the study	
☒	☐	Antibodies
☒	☐	Eukaryotic cell lines
☒	☐	Palaeontology and archaeology
☒	☐	Animals and other organisms
☐	☒	Human research participants
☒	☐	Clinical data
☒	☐	Dual use research of concern

Methods

n/a	Involved in the study	
☒	☐	ChIP-seq
☒	☐	Flow cytometry
☐	☒	MRI-based neuroimaging

Human research participants

Policy information about studies involving human research participants

Population characteristics	All participants were right-handed, native English speakers with normal to corrected-to-normal vision. As per the exclusion criteria, none of the participants had any prior history of psychiatric or neurological disorders, incompatibility for MRI scanning, severe claustrophobia and anticipated pregnancy or drug use that could alter cognitive functioning. Consequently, the final group for Experiment 1 consisted of 46 participants (mean = 21.31 years old, SD = 2.17, range = 18-29, 29/17 female to male ratio), whereas the final number of participants included in Experiment 2 was 140 (mean = 20.70 years old, SD = 2.37, range = 18-31, 83/57 female to male ratio), who fully completed all the required neuroimaging-based and behavioural assessments.
Recruitment	All participants for both experiments were recruited from the undergraduate and postgraduate population at the University of York via online and poster advertisements. All volunteers received monetary compensation or course credit for their participation in line with the departmental policies. There may be a selection bias given that participants who agreed to take part in our experiments are generally young adults and are enrolled in higher education. Although, we do not expect this to have a significant influence on our results, future studies with more representative samples of the population will be required to assess the generalisability of our findings.
Ethics oversight	For both Experiment 1 and 2, ethical approval was obtained from the Department of Psychology and York Neuroimaging Centre, University of York ethics committees. All participants were briefed about the aims and objectives of the experiments before providing informed consent to take part in this study.

Note that full information on the approval of the study protocol must also be provided in the manuscript.

Magnetic resonance imaging

Experimental design

Design type	Experiment 1 included two task-based, event-related fMRI studies that were repeated within same participants. Experiment 2 was an individual difference study that employed resting state fMRI.
Design specifications	In Experiment 1, semantic and episodic memory retrieval was assessed using 3-alternative forced-choice (3-AFC) paradigms. Both tasks consisted of 40 strong association, 40 weak association and 20 control trials. Following the presentation of the probe, target and 2 distractors, participants were given 4 seconds to respond, after which a fixation cross was displayed, jittered in duration between 1.5 to 3.5 seconds in 500 ms intervals.
Behavioral performance measures	For all task-based paradigms, both accuracy and response times were recorded. The mean inverse efficiency scores (i.e. reaction time divided by the percentage of incorrect responses) were calculated for each participant, the mean and standard deviation of which were used as indicators of compliant performance.

Acquisition

Imaging type(s)	Functional and Structural MRI
Field strength	3T
Sequence & imaging parameters	The high-resolution structural scans across both experiments were obtained with sagittal 3D fast spoiled gradient-recalled echo T1-weighted imaging (TR = 7.8 s, TE = 3 ms, flip angle = 20°, FOV = 289 x 289 mm2, matrix size = 256 x 256, voxel size = 1.13 x 1.13 x 1 mm, 176 slices). The functional MRI data for both semantic and episodic 3-AFC tasks as well as resting state scanning was collected using a single-shot 2D gradient-echo-planar imaging sequence (TR = 3.0 s, TE = 18.9 ms, flip angle = 90°, FOV = 192 x 192 mm2, matrix size = 64 x 64, voxel size = 3 x 3 x 3 mm3, 60 axial slices with no gap and slice thickness of 3 mm). For the task-based functional MRI, scan durations were 10.83 minutes (217 volumes) each, whereas for resting state functional MRI the duration was 9 minutes (180 volumes).
Area of acquisition	The field of view employed in the MRI scans covered the whole brain.

Diffusion MRI ☐ Used ☒ Not used

Preprocessing

Preprocessing software	FSL (Version 5.0.11) was used to pre-process the task-based MRI data. SPM (Version 12.0) and CONN Toolbox (Version 17.f) was used for the resting state MRI data.
Normalization	Task-based (Experiment 1): After coregistration to the structural images, individual functional images were linearly registered to the MNI-152 template using FMRIB's Linear Image Registration Tool (FLIRT). Resting state (Experiment 2): Structural images were coregistered to the mean functional image via rigid-body transformation, segmented into grey/white matter and cerebrospinal fluid probability maps, and images were spatially normalized to the MNI-152 template using the unified segmentation-normalization procedure.
Normalization template	MNI-152.
Noise and artifact removal	Task-based (Experiment 1): The individual subject analysis first involved motion correction using MCFLIRT and slice-timing correction using Fourier space time-series phase-shifting. Functional images were spatially smoothed using a Gaussian kernel of FWHM 5mm, underwent grand-mean intensity normalization of the entire 4D dataset by a single multiplicative factor, and both high-pass temporal filtering (Gaussian-weighted least-squares straight line fitting, with sigma=100s) and Gaussian low-pass temporal filtering, with sigma=2.8s. For all general linear models (GLMs), standard motion parameters (3 rotations and translations), their temporal derivatives and squared versions were added as potential motion confounds. No significant difference in head-motion was observed between the two task conditions. Resting state (Experiment 2): An extensive set of motion-correction and denoising procedures were employed, comparable to those reported in the literature (Ciric et al. 2017). In addition to the removal of six realignment parameters and their second-order derivatives using a GLM, a linear detrending term was applied as well as the CompCor method that removed five principal components of the signal from white matter (WM) and cerebrospinal fluid (CSF). The composite motion score (i.e. percentage of invalid scans) for each participant was also added as a covariate in group-level analyses to further account for the potential influence of head motion on functional connectivity estimates. No significant correlation was observed between in-scanner head motion and covariates of interest utilised in subsequent analyses.
Volume censoring	Task-based (Experiment 1): Motion outliers were identified using the DVARS method and included in the model as a covariate of no-interest. Resting-state (Experiment 2): The functional volumes influenced by excessive head motion were identified and scrubbed based on the conservative settings of motion greater than 0.5 mm and global signal change larger than z = 3. Participants who had more than 15% of their data affected by motion were excluded from further analysis.

Statistical modeling & inference

Model type and settings	Task-based (Experiment 1): The pre-processed task fMRI data was modelled using mass univariate general linear models (GLM) with the onsets and durations (4s) of all trial types (i.e. strong, weak, control) included at the subject-level. Group-level statistical contrasts of strong > weak, weak > strong and task (all trials) > control was assessed using a mixed-effects approach

with FLAME. Conjunction analyses across semantic and episodic tasks were carried out with the easythresh_conj command from FSL that relies on the minimum statistic of the conjunction null. Resting-state (Experiment 2): For each participant, average BOLD timeseries obtained from the binarized version of the significant control cluster from Experiment 1 was correlated with time courses from the rest of the brain in order to obtain individual and group average connectivity maps. Group-level linear regression with seed-based connectivity was performed in which inverse efficiency scores across both semantic and episodic memory tasks were included as the independent variables of interest. Age, gender and composite motion score (i.e. percentage of invalid scans identified during the scrubbing procedure) were added as nuisance variables in the model.

Effect(s) tested

Task-based (Experiment 1): Using both repeated-measures and one-sample t-test designs, Experiment 1 assessed brain activity related to the processing of strong versus weak trials across both 3-AFC semantic and episodic memory retrieval tasks, and the interaction between all memory retrieval versus control trials and semantic versus episodic memory retrieval, respectively. A conjunction analysis was employed to test common set of brain regions that showed shared responses across both semantic and episodic memory retrieval tasks.

Specify type of analysis: ☐ Whole brain ☐ ROI-based ☒ Both

Anatomical location(s)

A seed-based functional connectivity analysis was carried out in Experiment 2, based on a significant cluster of brain regions identified in Experiment 1.

Statistic type for inference
(See Eklund et al. 2016)

Group level analyses were carried out using a cluster-forming threshold of Z > 2.6 (p < .005 at the voxel level).

Correction

Whole-brain FWE cluster correction was employed at p < .05.

Models & analysis

n/a	Involved in the study
☐	☒ Functional and/or effective connectivity
☒	☐ Graph analysis
☐	☒ Multivariate modeling or predictive analysis

Functional and/or effective connectivity

Resting state (Experiment 2): The seed-based functional connectivity approach was based on bivariate Pearson correlations between average signal from the chosen seed ROI and all other voxels across the whole-brain.

Multivariate modeling and predictive analysis

Resting state (Experiment 2): Multivariate analysis of variance (MANOVA) approach was employed with the Pearson correlation between the chosen seed-region and the rest of the brain entered as the dependent variable, inverse efficiency scores across participants as the independent variable and age, gender, head-motion inside the scanner as covariates of no-interest.
